# Postoperative radiotherapy for completely resected thymoma and thymic carcinoma: A systematic review and meta-analysis

**DOI:** 10.1371/journal.pone.0308111

**Published:** 2024-08-30

**Authors:** Tianyu He, Jiacheng Yao, Jun Chen, Tingting Liu, Jun Dang

**Affiliations:** 1 Department of Radiation Oncology, The First Hospital of China Medical University, Shenyang, China; 2 Department of Radiation Oncology, Shenyang Tenth People’s Hospital, Shenyang, China; 3 Department of Radiation Oncology, Anshan Cancer Hospital, Anshan, China; University of Leeds, UNITED KINGDOM OF GREAT BRITAIN AND NORTHERN IRELAND

## Abstract

**Background:**

The role of postoperative radiotherapy (PORT) after complete tumor resection in patients with thymoma or thymic carcinoma remains controversial. We performed a meta-analysis to identify groups that would benefit from PORT.

**Methods:**

Multiple scientific databases were systematically searched for studies comparing overall survival (OS) and/or disease-free survival (DFS) between PORT and surgery alone in patients with completely resected thymomas or thymic carcinomas until April 10, 2024. A random-effects model was used for the statistical analysis.

**Results:**

A total of 31 studies with 10543 patients were included (17 studies involving 4763 patients with thymoma, seven studies involving 1045 patients with thymic carcinoma, and seven studies involving 4735 patients with mixed histological types). Notably, PORT significantly prolonged OS (hazard ratio [HR] = 0.73, 95% confidence interval [CI]: 0.59–0.91) and DFS (HR = 0.62, 95% CI: 0.43–0.89). Similar results were also observed when the multivariate-adjusted HRs were used as the measure of effect (OS: HR = 0.60, 95% CI: 0.43–0.83; DFS: HR = 0.48, 95% CI: 0.29–0.79). In subgroup analyses, PORT was associated with a longer OS and DFS for thymoma (HR = 0.73, 95% CI: 0.56–0.96 and HR = 0.65, 95% CI: 0.46–0.93), thymic carcinoma (HR = 0.72, 95% CI: 0.49–1.07 and HR = 0.38, 95% CI: 0.19–0.77), and stage 3–4 disease (HR = 0.50, 95% CI: 0.34–0.74 and HR = 0.44, 95% CI: 0.27–0.70), but not for stage 2 disease (HR = 0.81, 95% CI: 0.55–1.19 and HR = 0.97, 95% CI: 0.51–1.83).

**Conclusions:**

PORT is likely to improve OS and DFS in patients with completely resected stage 3–4 thymoma or thymic carcinoma; however, the value of PORT for stage 2 disease requires further evaluation in large-scale studies.

## Introduction

Thymic epithelial tumors (TETs) are rare tumors with an incidence of 3.2 cases per million [1], and are classified as thymoma, thymic carcinoma, and thymic neuroendocrine tumors. Surgery is the mainstay of treatment for resectable thymoma or thymic carcinoma, and complete tumor resection is the most significant predictor of outcomes [2, 3]. Postoperative radiotherapy (PORT) is considered important for incompletely resected cases. However, the value of PORT in patients with complete tumor resection remains controversial owing to the lack of randomized controlled trials and conflicting results from observational studies [4–34] and meta-analyses [35–38].

In fact, completely resected TETs are heterogeneous. Notably, PORT has been associated with improved survival in patients with stage 3 but not stage 2 thymoma [31]. Patients with thymoma with histologic subtype B benefited more from PORT than those with histologic subtype A [19], and PORT using three-dimensional conformal radiation therapy (3D-CRT) or intensity-modulated radiation therapy (IMRT) had a higher 5-year OS rate than two-dimensional radiation therapy (2D-RT) [11]. These findings suggest that the benefit of PORT appears to be affected by the clinicopathological features of patients, and differences in these features in individual studies may account for the inconsistent findings. To date, few studies have specifically addressed the role of PORT based on the clinicopathological features of patients. Therefore, individualized assessment of the value of PORT remains essential.

In light of these issues, we conducted a comprehensive meta-analysis of the currently available evidence on completely resected TETs to clarify the subgroup of patients who could benefit from PORT.

## Methods

### Literature search

This study was conducted in accordance with the Preferred Reporting Items for Systematic Reviews and Meta-Analyses (PRISMA) 2020 statement [39]. Two authors (HT and YJ) independently searched PubMed, Embase, Cochrane Library, and Web of Science databases as of April 10, 2024. The detailed search strategies are shown in S1 Table. Furthermore, references in the relevant reviews were manually checked.

### Inclusion and exclusion criteria

The inclusion criteria were as follows: (1) prospective or retrospective study design; (2) study population comprising patients with completely resected (R0) thymoma and/or thymic carcinoma; (3) interventions comprising PORT and surgery alone; (4) outcomes including overall survival (OS), disease-free survival (DFS), and/or recurrence-free survival (RFS); and (5) publication in English. Studies that included a small proportion of patients with incomplete tumor resection (<10%) were also considered eligible. In case of overlap of patient data among the studies, the study with the most comprehensive data was selected.

### Data extraction and quality assessment

Two authors (HT and YJ) independently extracted the following data from each article: name of the first author; year of publication; period of recruitment; study design; median follow-up time; sample size in each group; and hazard ratios (HRs) with 95% confidence intervals (CIs) for OS, DFS, and RFS.

The Newcastle-Ottawa scale [40] was used to evaluate the quality of the studies, and the Grading of Recommendations Assessment, Development, and Evaluation (GRADE) [41] was used to assess the quality of the evidence.

### Statistical analysis

This meta-analysis was conducted using R software (version 3.5.3, R Foundation for Statistical Computing) via the meta-package using the random-effects model. The outcomes of interest were OS, DFS, and RFS, which are presented as HRs with 95% CIs. Notably, HRs with 95% CIs were calculated using Kaplan–Meier curves [42] when not directly reported in the studies. The I^2^ test was used to investigate heterogeneity. Univariate and multivariate meta-regression analyses were performed to assess sources of heterogeneity. Subgroup analyses were conducted according to the histological type, stage, year of publication, and region. The robustness of the results was evaluated using sensitivity analysis. Publication bias was estimated using funnel plots and Egger’s test [43].

## Results

### Literature search and study selection

**Fig 1:**
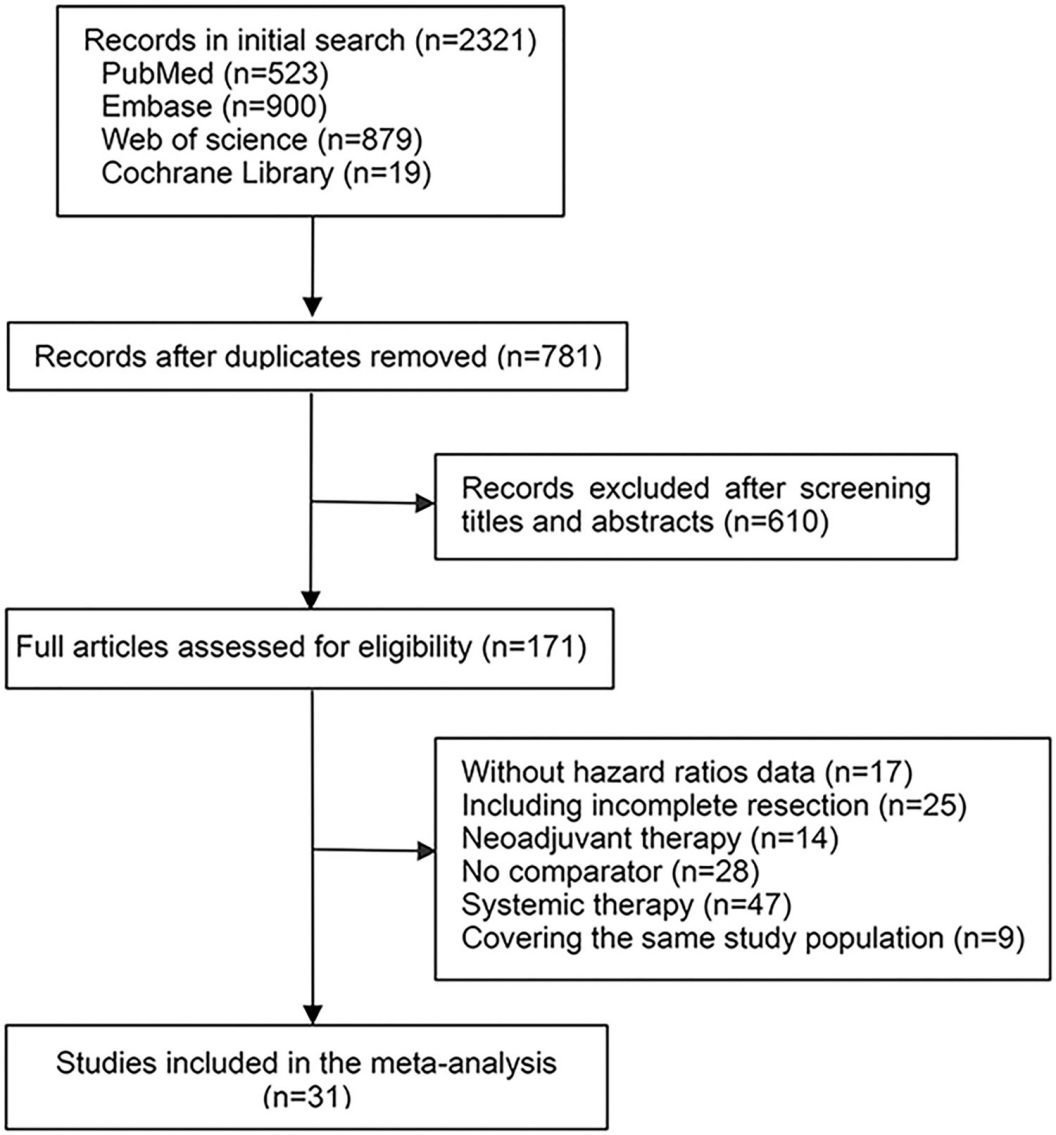
Literature search and selection.

Notably, 2321 records were found during the initial search. After removing duplicates, 781 records were identified. Of these, 610 publications were excluded after reviewing the titles and abstracts, and the remaining 171 articles underwent a full-text assessment. Fig 1 shows the study selection process and reasons for exclusion. Finally, 31 retrospective studies [4–34] with 10543 patients (4945 who underwent PORT and 5598 who underwent surgery alone), were included in this meta-analysis. Among them, there were 17 studies involving 4763 patients with thymoma, seven studies involving 1045 patients with thymic carcinoma, and seven studies involving 4735 patients with mixed histological types. The Masaoka or Masaoka-Koga staging system was used in all studies. Six studies [17–19, 22, 27, 29] included a small number of patients with incomplete tumor resection (<10% in each study). Most studies did not provide detailed information regarding the surgical method, RT technique, RT dose, or neoadjuvant or adjuvant chemotherapy. Characteristics of the included studies are listed in Table 1.

**Table 1:** Characteristics of included studies.

First author/ year	Region	Study period	Median follow-up (months)	Age (median)	Sex (males, %)	Histological type	Stage	R0 resection (%)	No. of patients (PORT/surgery alone)
Regnard/1996 [4]	France	1955–1993	96	NR	NR	Thymoma	2–3	100	90/24
Singhal/2003 [5]	USA	1992–2002	70	70	44	Thymoma	1–2	100	23/47
Kondo/2003 [6]-C	Japan	1990–1994	NR	NR	NR	Carcinoma	1–4	100	33/16
Kondo/2003 [6]-T	Japan	1990–1994	NR	NR	NR	Thymoma	3–4	100	105/35
Rena/2007 [7]	Italy	1988–2000	91	51	52	Thymoma	2	100	26/32
Chen/2010 [8]	China	1964–2006	63	47	50	Thymoma	2	100	66/41
Chang/2011 [9]	Korea	1988–2009	59	52	53	Thymoma	2–3	100	59/17
Weksler/2012 [10]	USA	1973–2007	NR	57	56	Thymoma	3	100	322/154
Fan/2013 [11]	China	1982–2010	50	NR	51	Thymoma	3	100	53/12
Shen/2013 [12]	China	2001–2006	84	NR	NR	Mixed	1–4	100	33/50
Song/2014 [13]	China	1995–2009	NR	NR	NR	Carcinoma	2	100	21/10
Sakamaki/2014 [14]	Japan	1998–2011	49	NR	NR	Thymoma	1–2	100	10/72
Ruffini/2014 [15]	Italy	1990–2010	48	NR	50	Mixed	1–4	100	676/986
Mao/2015 [16]	China	2001–2013	72	49	48	Carcinoma	1–3	100	35/29
Omasa/2015 [17]-C	Japan	1991–2010	56	60	63	Carcinoma	2–3	91	80/75
Omasa/2015 [17]-T	Japan	1991–2010	56	58	43	thymoma	2–3	98	323/787
Liu/2016 [18]	China	1994–2012	NR	51	53	Mixed	1–3	90	649/897
Rimner/2016 [19]	USA	1990–2012	NR	54	50	thymoma	2–3	100	689/574
Fu/2016 [20]	China	1996–2013	36	52	67	Carcinoma	1–4	100	211
Hishida/2016 [21]	Japan	1991–2010	104	NR	NR	Carcinoma	1–4	100	79/90
Narm/2016 [22]	Korea	2000–2013	NR	NR	NR	Thymoma	1–2	97	288/474
Jackson/2017 [23]	USA	2004–2012	57	NR	47	Thymoma	1–4	100	101/149
Lim/2017 [24]	USA	2004–2013	NR	NR	57	Carcinoma	1–4	100	61/61
Yuan/2017 [25]	China	2003–2014	86	NR	NR	Thymoma	1–4	100	142/165
Liao/2018 [26]	China	2003–2013	70	NR	50	Mixed	3	100	74/25
Song/2020 [27]	Korea	2000–2013	NR	52	48	Thymoma	2–3	91	202/202
Kim/2020 [28]	USA	2004–2013	NR	NR	NR	Carcinoma	2b	100	56/33
Ak/2021 [29]	Turkey	2002–2018	198	49	53	Thymoma	2–4	95	76/123
Tang/2021 [30]-T	China	1988–2017	60	NR	NR	Thymoma	3	100	34/7
Tang/2021 [30]-C	China	1988–2017	48	NR	NR	Carcinoma	3	100	40/9
Zhou/2022 [31]-stage2	China	1991–2019	85	55	47	Thymoma	2	100	49/76
Zhou/2022 [31]-stage3	China	1991–2019	85	55	47	Thymoma	3	100	34/29
Chen/2023 [32]	China	2011–2021	NR	44	49	Thymoma	1–4	100	72/54
An/2023 [33]	China	2011–2021	NR	NR	NR	Thymoma	1–4	100	38/38
Rimner/2023 [34]	Multi	1990–2013	35	56	64	Carcinoma	1–4	100	201/99

PORT, postoperative radiotherapy; NR, not reported.

### Assessment of study and publication bias

Three studies [6, 14, 22] were rated with a score of 5, and the other studies had a score of ≥6 (S2 Table). No significant publication bias was observed in OS (Egger’s test, P = 0.61) or PFS (Egger’s test, P = 0.15). Funnel plots are shown in S1 Fig.

### PORT vs. surgery alone on OS, DFS, and RFS

**Fig 2:**
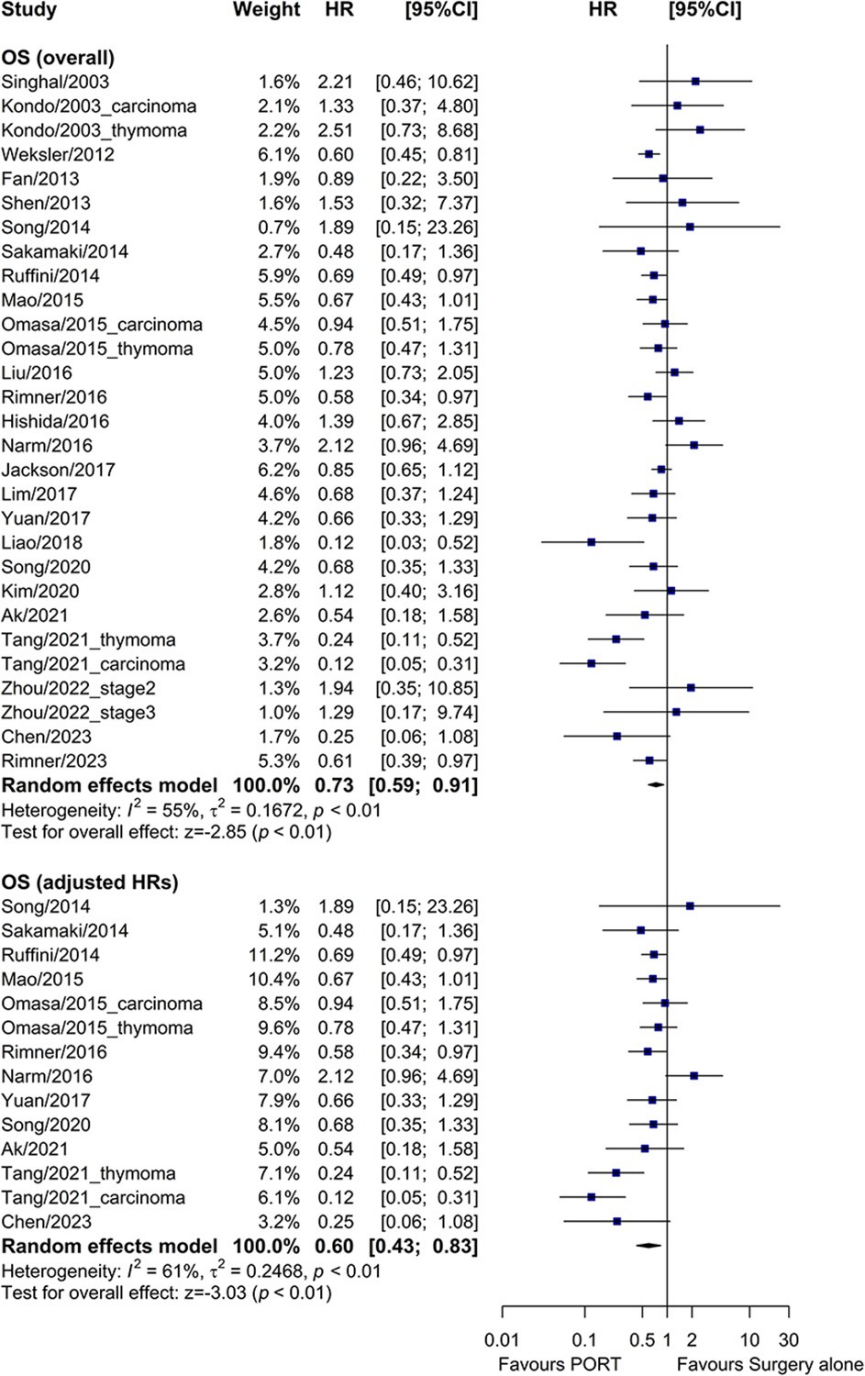
Forest plot of HRs comparing OS between the PORT and surgery alone groups. OS, overall survival; PORT, postoperative radiotherapy; HR, hazard ratio; CI, confidence interval.

**Fig 3:**
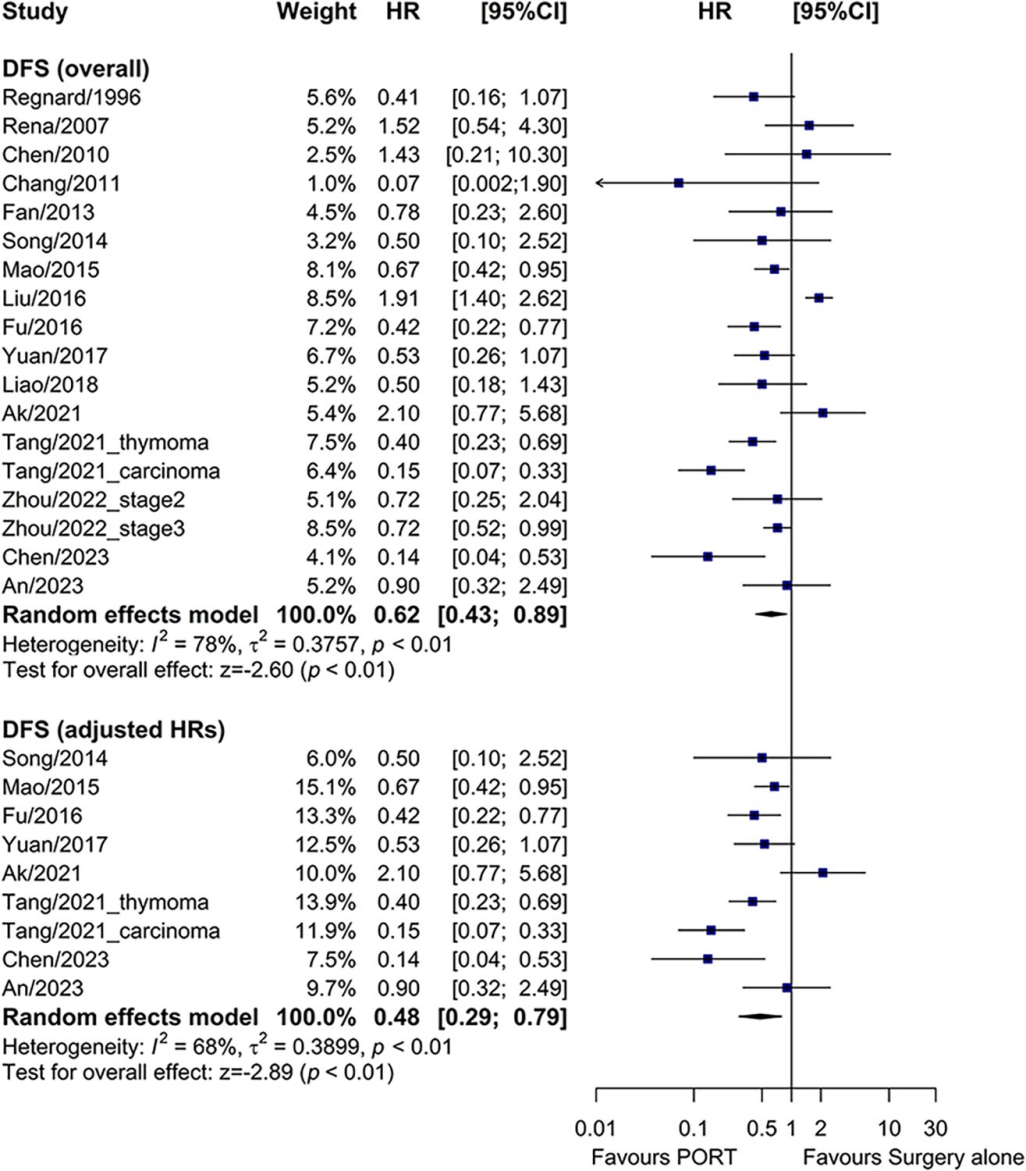
Forest plot of HRs comparing DFS between the PORT and surgery alone groups. DFS, disease-free survival; PORT, postoperative radiotherapy; HR, hazard ratio; CI, confidence interval.

Compared to surgery alone, PORT was associated with a significantly longer OS (HR = 0.73, 95% CI: 0.59–0.91, I^2^ = 55%) (Fig 2) and DFS (HR = 0.62, 95% CI: 0.43–0.89, I^2^ = 78%) (Fig 3), and a trend of better RFS (HR = 0.66, 95% CI: 0.43–1.01, I^2^ = 66%) (S2 Fig).

Twelve and seven studies reported multivariate-adjusted HRs for OS and DFS, respectively. When these adjusted HRs were used as the measure of effect, PORT also significantly improved OS (HR = 0.60, 95% CI: 0.43–0.83, I^2^ = 61%) (Fig 2) and DFS (HR = 0.48, 95% CI: 0.29–0.79, I^2^ = 68%) (Fig 3).

### Cumulative meta-analysis of PORT vs. surgery alone on OS and DFS

**Fig 4:**
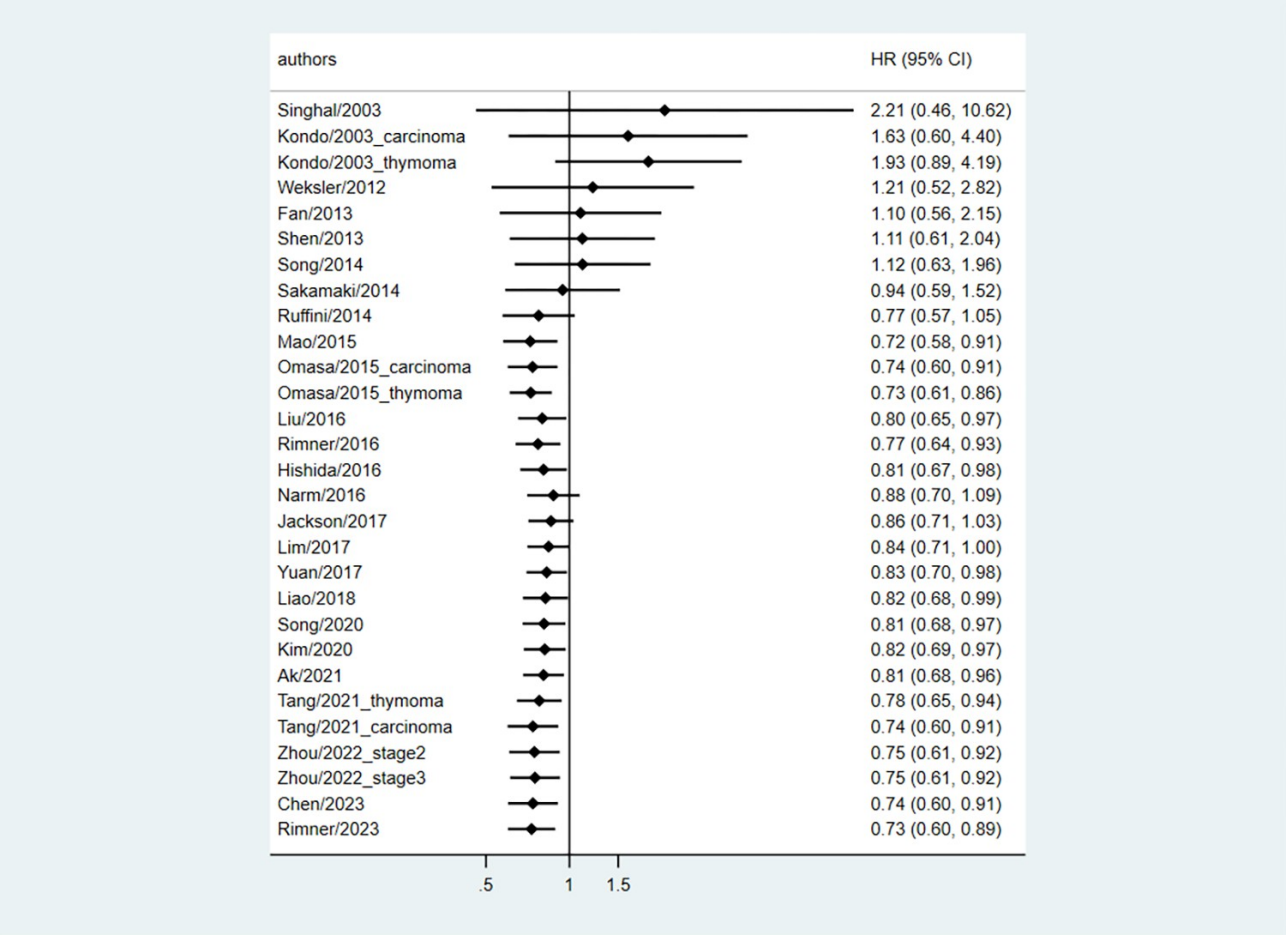
Cumulative meta-analysis of OS. OS, overall survival; HR, hazard ratio; CI, confidence interval.

Because the included studies had a time span of >20 years, we conducted a cumulative meta-analysis in which the studies were added in the order of publication year. We found that the OS benefit of PORT was generally observed in publications since 2015, but not in most publications before 2015 (Fig 4). The cumulative meta-analysis of DFS showed similar results (S3 Fig).

### Subgroup analysis of PORT vs. surgery alone on OS and DFS

**Fig 5:**
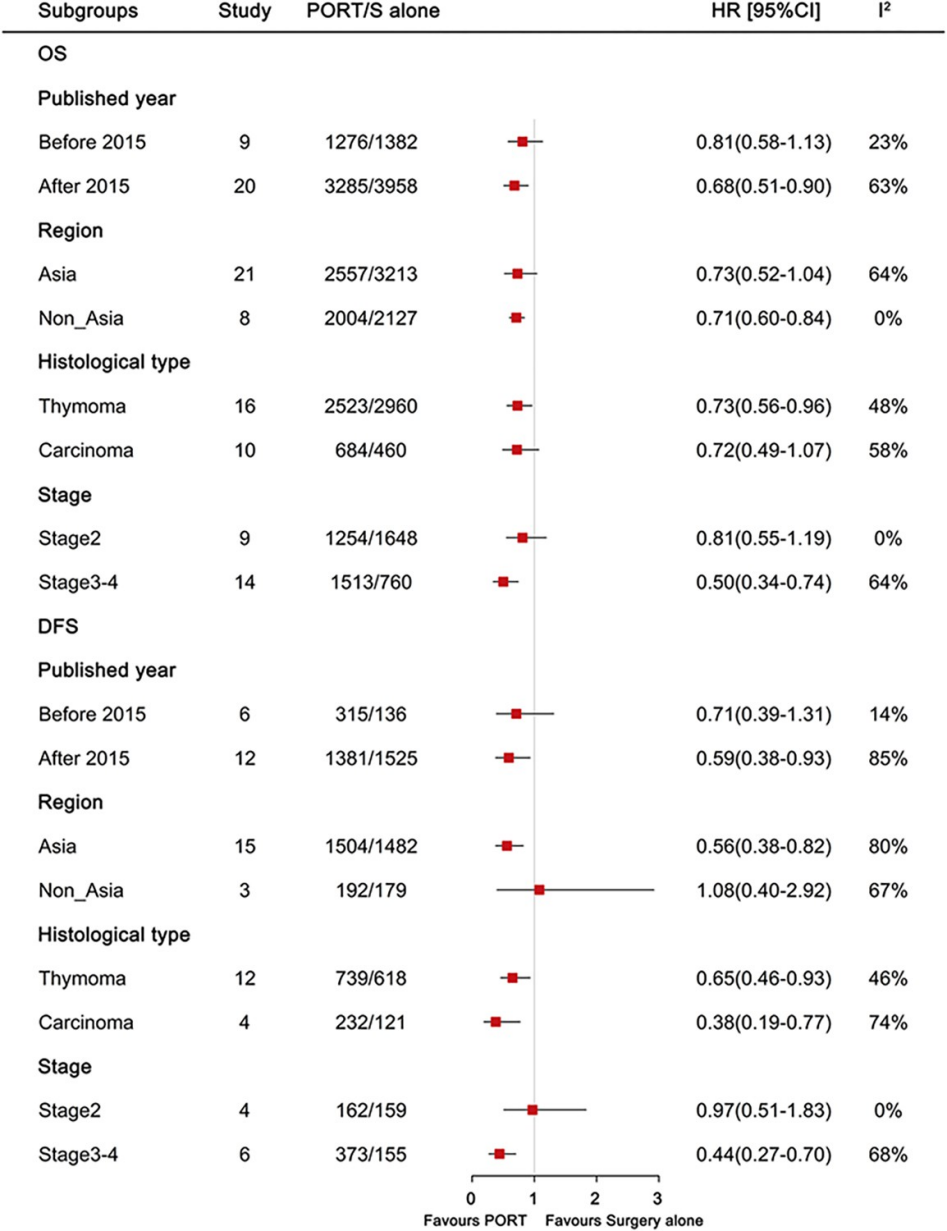
Subgroup analyses for OS and DFS. OS, overall survival; DFS, disease-free survival; PORT, postoperative radiotherapy; S, surgery; HR, hazard ratio; CI, confidence interval.

The results of the subgroup analyses are summarized in Fig 5, and the forest plot for each result can be found in S4–S11 Figs.

### Studies published by year

A significantly longer OS (HR = 0.68, 95% CI: 0.51–0.90, I^2^ = 63%) and DFS (HR = 0.59, 95% CI: 0.38–0.93, I^2^ = 85%) with PORT were observed in studies published since 2015, but not in studies published before 2015 (OS: HR = 0.81, 95% CI: 0.58–1.13, I^2^ = 23%; DFS: HR = 0.71, 95% CI: 0.39–1.31, I^2^ = 14%).

### Region

Notably, PORT achieved better OS for non-Asian populations (HR = 0.71, 95% CI: 0.60–0.84, I^2^ = 0%), and showed a trend of better OS for Asian populations (HR = 0.73, 95% CI: 0.52–1.04, I^2^ = 63%). Furthermore, DFS benefits from PORT were observed for Asian populations (HR = 0.56, 95% CI: 0.38–0.82, I^2^ = 80%), but not for non-Asian populations (HR = 1.08, 95% CI: 0.40–2.92, I^2^ = 67%).

### Histological type

Notably, PORT significantly prolonged OS (HR = 0.73, 95% CI: 0.56–0.96, I^2^ = 48%) and DFS (HR = 0.65, 95% CI: 0.46–0.93, I^2^ = 46%) in patients with thymoma. Regarding patients with thymic carcinoma, PORT was associated with a significantly longer DFS (HR = 0.38, 95% CI: 0.19–0.77, I^2^ = 74%) and a trend of longer OS (HR = 0.72, 95% CI: 0.49–1.07, I^2^ = 58%).

### Stage

Furthermore, PORT showed longer OS and DFS in patients with stage 3–4 disease (HR = 0.50, 95% CI: 0.34–0.74, I^2^ = 64% and HR = 0.44, 95% CI: 0.27–0.70, I^2^ = 68%) than in patients with stage 2 disease (HR = 0.81, 95% CI: 0.55–1.19, I^2^ = 0% and HR = 0.97, 95% CI: 0.51–1.83, I^2^ = 0%).

### The GRADE assessment

The results of the GRADE assessment are presented in S3 Table. The evidence for OS, DFS, and RFS showed low GRADE ratings. Regarding the subgroup analyses, the evidence for OS and/or PFS had moderate GRADE ratings in the subgroups of adjusted HRs, publications before 2015, non-Asian populations, thymoma, and thymic carcinoma and low GRADE ratings in the other subgroups.

### Meta-regression analysis

Univariate and multivariate meta-regression analyses were conducted to investigate whether age, sex, year of publication, region, histological type, and stage were sources of heterogeneity (S4 Table). Multivariate meta-regression showed that publication year and stage were significantly associated with OS heterogeneity (P = 0.03 and P = 0.007, respectively).

### Sensitivity analysis

Sensitivity analysis for OS and DFS showed that omitting one study at a time did not significantly alter the results (S12 Fig).

## Discussion

This meta-analysis comprehensively assessed the role of PORT in patients with completely resected thymoma and thymic carcinoma. Our results showed that PORT significantly improved OS and DFS compared to surgery alone. Similar results were observed when multivariate-adjusted HRs were used as a measure of the effect. However, in the subgroup analysis, OS and PFS benefits with PORT were observed in stage 3–4 disease, but not in stage 2 disease.

Although the current National Comprehensive Cancer Network guidelines recommend the use of PORT for completely resected stage 2–4 thymoma and thymic carcinoma [44], few clinical studies have demonstrated the survival benefit of PORT in patients with stage 2 disease. The heterogeneity of stage 2 tumors may affect the efficacy of PORT. In a study using the International Thymic Malignancy Interest Group database [19], PORT significantly prolonged OS for completely resected stage 2 thymomas, and histologic subtypes B1–B3 were associated with the greatest OS benefit. Another study based on the National Cancer Data Base [23] showed significantly improved OS with PORT in patients with stage 2B thymoma, but not in those with stage 2A thymoma. These findings suggest that PORT may be suitable for selected stage 2 patients, such as those with extensive capsular invasion (stage 2B) and/or aggressive histological subtypes (B1–3). However, these hypotheses must be validated in large-scale studies.

Given that thymic carcinoma is more aggressive, PORT should be more useful in patients with this histological type. In two single-institution studies [16, 29], PORT achieved better OS and DFS in patients with stage 1–3 thymic carcinoma after complete resection. Significantly improved DFS and a trend towards better OS with PORT in patients with thymic carcinoma were also observed in our meta-analysis. Nevertheless, two studies based on the Japanese Association for Research of the Thymus [20] and the Surveillance, Epidemiology, and End Results [24] databases did not demonstrate an OS benefit of PORT for completely resected thymic carcinoma. Notably, the use of adjuvant chemotherapy in individual studies was inconsistent. Some studies have demonstrated the survival benefits of adjuvant chemotherapy for completely resected thymic carcinoma [29, 45]. In a study by Tang et al. [30], adjuvant chemotherapy was associated with improved OS in thymic carcinoma with superior vena cava or innominate vein invasion. In another study by Gao et al. [45], the most common failure was distant metastasis in patients with stage 3–4 thymic carcinoma after complete resection, and PORT combined with adjuvant chemotherapy (POCRT) was an independent predictor of improved DFS. These results indicate that PORT may be insufficient, and POCRT should be considered for completely resected thymic carcinomas, especially in patients with advanced-stage disease.

Considering the patients recruited in studies spanning more than six decades, we conducted a cumulative meta-analysis according to the year of publication. We found that publications since 2015 generally reported significantly better OS in the PORT group, whereas most publications before 2015 did not. Subgroup analysis according to the year of publication also showed significantly improved OS and DFS with PORT in studies published after 2015, but not in studies published before 2015. One possible reason for these findings is the increased use of modern RT techniques, such as 3D-RT or IMRT, over time. Fan et al. [11] demonstrated a higher 5-year OS (100% vs. 86.9%) and lower regional recurrence (3.6% vs. 32%) rates with PORT using 3D-CRT/IMRT vs. 2D-RT in patients with completely resected stage 3 thymoma. Although detailed information on RT techniques was not provided in most of the studies included in our meta-analysis, more patients were likely to receive 3D-RT/IMRT in studies published after 2015 than in those published before 2015 according to the study period.

Overall, our results support the use of PORT in a subgroup of patients with stage 3–4 thymoma or thymic carcinoma. Although significantly positive results were not observed for stage 2 disease, some selected cases (such as stage 2B and histological subtype B) seemed to benefit from PORT. The use of 3D-CRT/IMRT is possibly an important factor associated with improved survival after PORT. Despite requiring further validation, these findings will be helpful to clinicians in developing individual treatment strategies for this patient population.

Differing from our results, two previously published meta-analyses [36, 38] found an OS benefit of PORT in patients with resected stage 2 thymomas or thymic carcinomas. However, the meta-analysis by Zhou et al. [36] (14 included studies) included many patients with incomplete or unclear resection statuses. Although a meta-analysis by Tateishi et al. [38] used adjusted HRs as a measure of effect, only five studies were included in their meta-analysis, with only two studies on stage 2 disease. Therefore, drawing conclusions regarding the benefits of PORT in patients with stage 2 tumors remains difficult.

The strengths of the present study are the large number of studies and sample size, and the fact that almost all of the patients included in the study underwent complete resection. In addition, considering the imbalance in background characteristics between the treatment groups, adjusted HRs were used as a measure of effect to validate the results. Moreover, comprehensive subgroup analyses were performed. All of these would be helpful in increasing the statistical power of our analyses.

However, our study has some limitations. First, all data in our meta-analysis were collected from retrospective studies, which have inherent limitations such as selection biases. Second, the heterogeneity was high for OS and DFS. The results of the meta-regression analyses indicated that disease stage was significantly associated with heterogeneity. In addition, histological subtype, adjuvant chemotherapy, RT technique, and surgical method may also be confounding factors. Third, adverse events could not be assessed because most included studies did not provide this information. Finally, some HRs were calculated using the Kaplan-Meier curve, which might result in an error in comparison with the direct calculation from the raw data.

## Conclusions

Our results suggest that PORT is likely to improve OS and DFS in patients with completely resected stage 3–4 thymoma or thymic carcinoma; however, the value of PORT for stage 2 disease requires further evaluation in large-scale studies. These findings should be validated in future large-scale studies. In addition, the roles of POCRT and PORT using modern RT techniques should be assessed in future studies.

## Supporting information

S1 Checklist PRISMA 2020 checklist. (DOCX)

S1 Fig Funnel plots of publication bias. (TIF)

S2 Fig The forest plot for recurrence-free survival. (TIF)

S3 Fig Cumulative meta-analysis for disease-free survival. (TIF)

S4 Fig The forest plot of overall survival for subgroup of study published year. (TIF)

S5 Fig The forest plot of disease-free survival for subgroup of study published year. (TIF)

S6 Fig The forest plot of overall survival for subgroup of study region. (TIF)

S7 Fig The forest plot of disease-free survival for subgroup of study region. (TIF)

S8 Fig The forest plot of overall survival for subgroup of histological type. (TIF)

S9 Fig The forest plot of disease-free survival for subgroup of histological type. (TIF)

S10 Fig The forest plot of overall survival for subgroup of stage. (TIF)

S11 Fig The forest plot of disease-free survival for subgroup of stage. (TIF)

S12 Fig Sensitivity analysis. (TIF)

S1 Table Search strategy. (DOC)

S2 Table Quality assessment of retrospective studies using the Newcastle-Ottawa scale. (DOC)

S3 Table GRADE assessment. (DOC)

S4 Table Meta-regression analysis. (DOC)
