# Supplementary figures and images for: Postoperative radiotherapy for completely resected thymoma and thymic carcinoma: A systematic review and meta-analysis
Source: PLoS One. 2024 Aug 30;19(8):e0308111. doi: 10.1371/journal.pone.0308111 (PMC11364254; doi:10.1371/journal.pone.0308111)

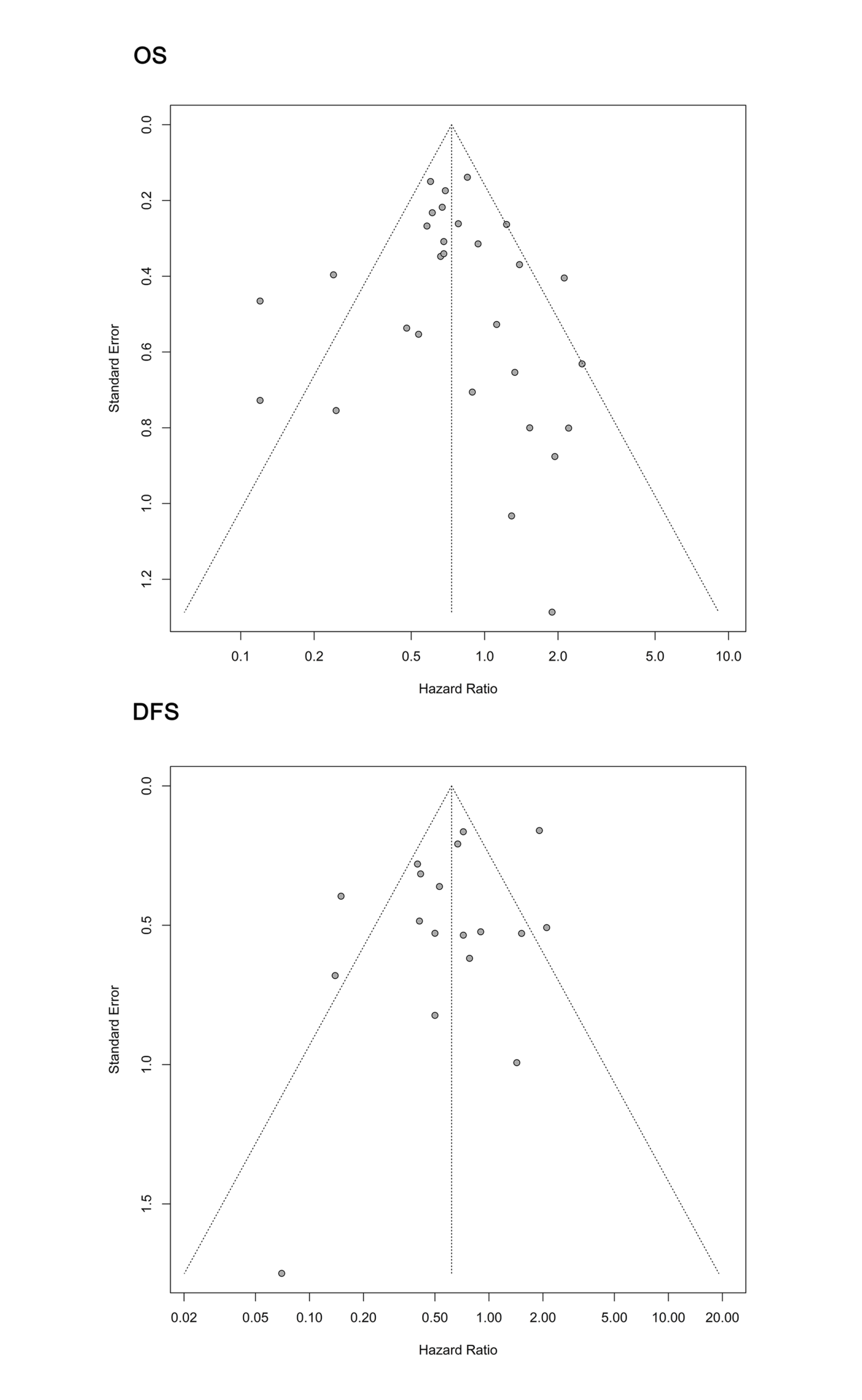

Supplement: S1 Fig — (TIF) [file pone.0308111.s002.tif]

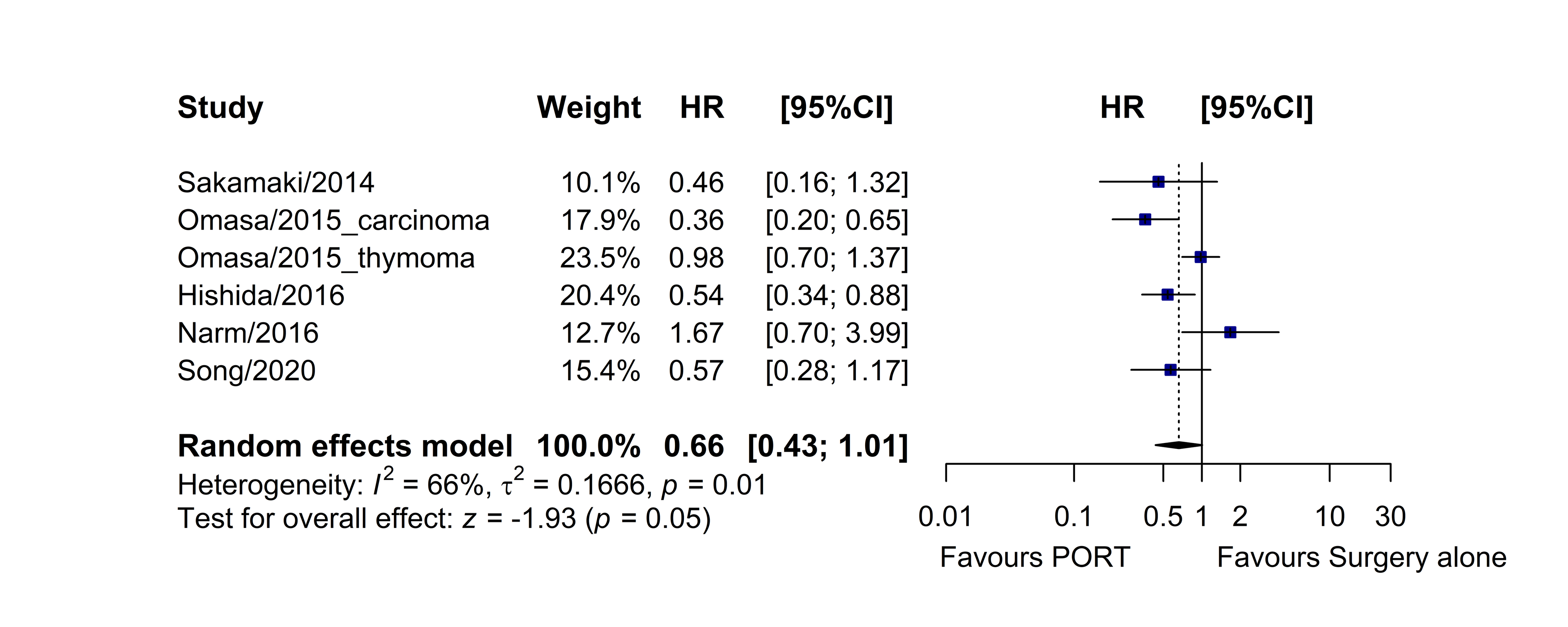

Supplement: S2 Fig — (TIF) [file pone.0308111.s003.tif]

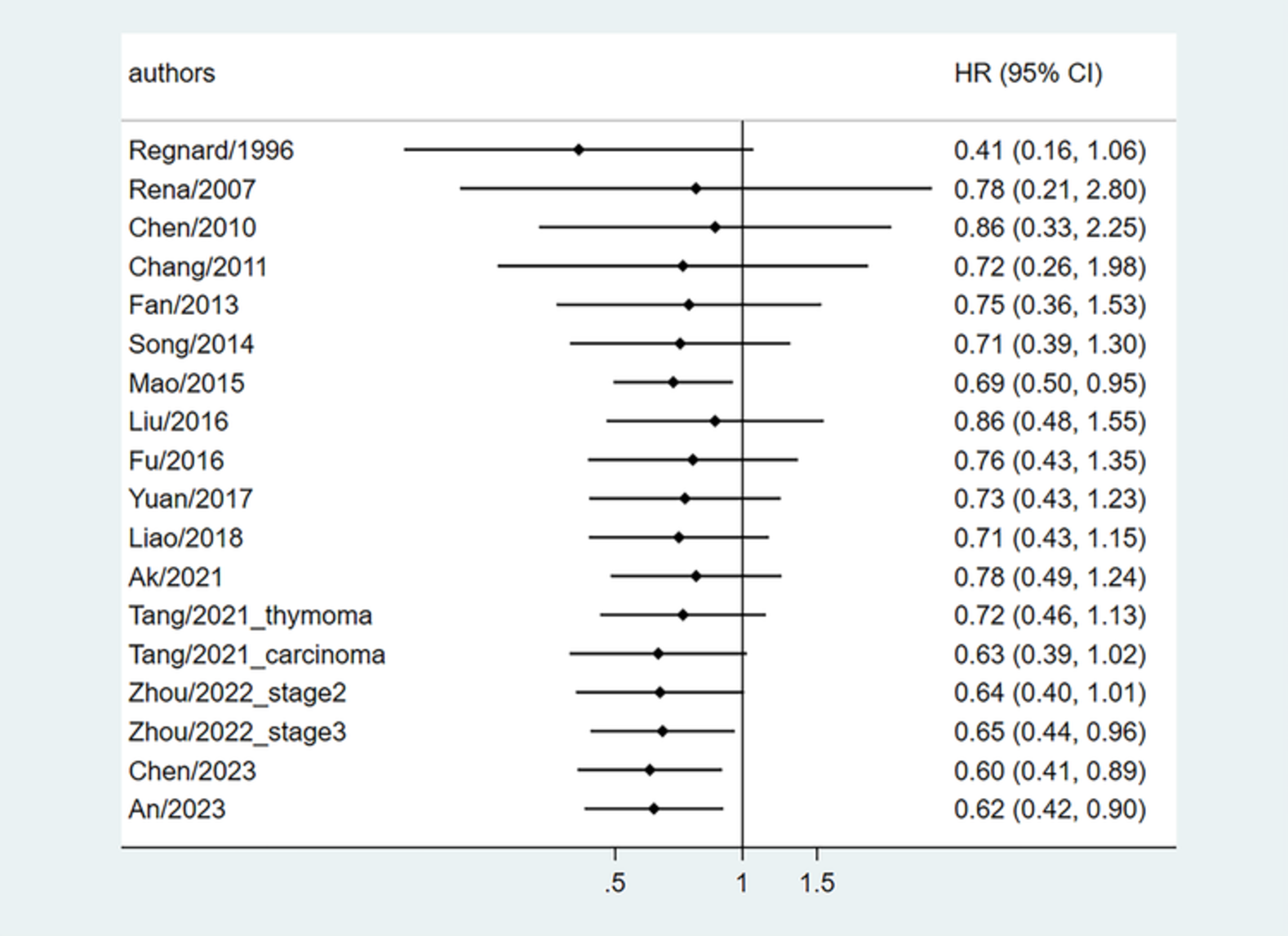

Supplement: S3 Fig — (TIF) [file pone.0308111.s004.tif]

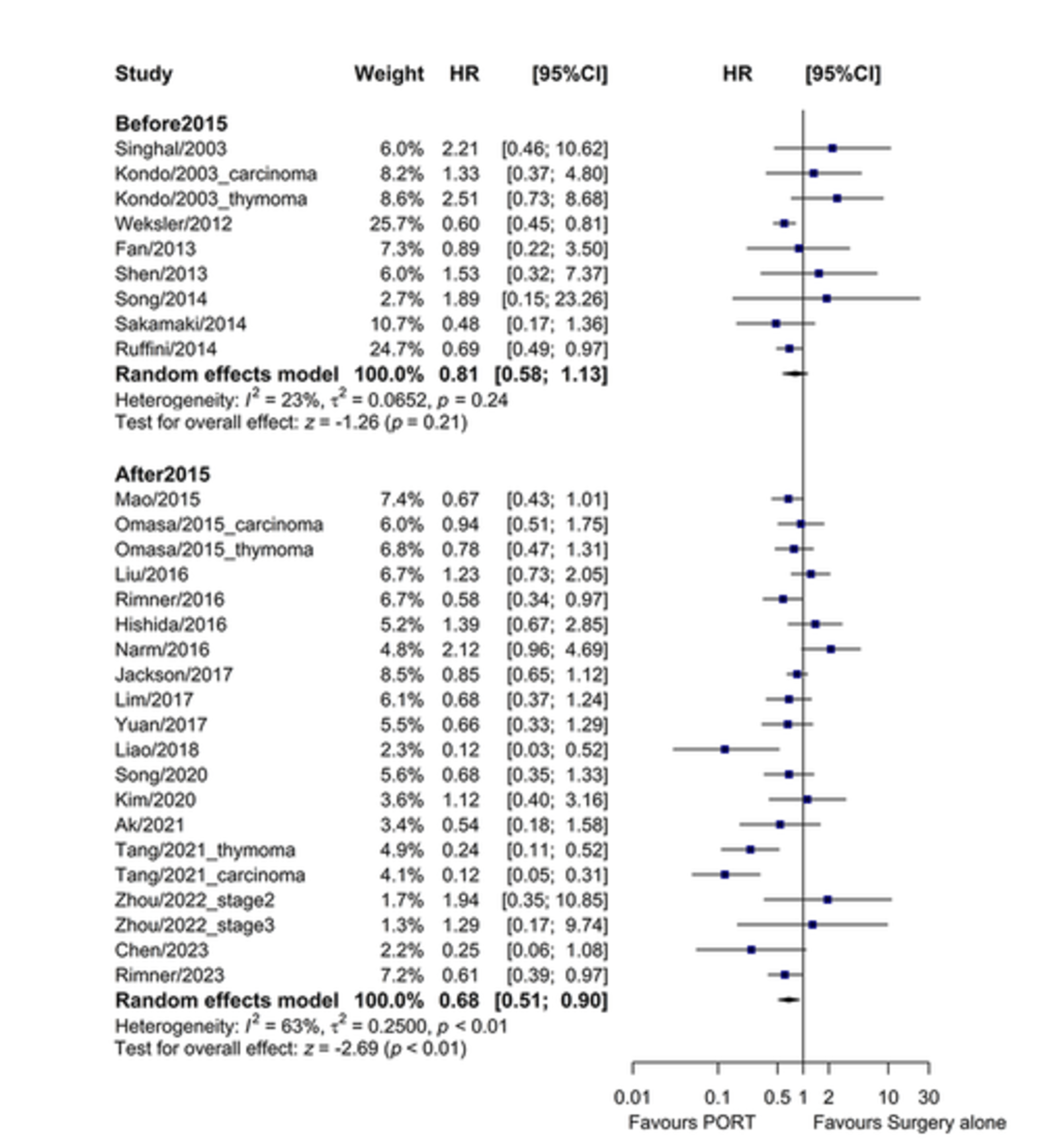

Supplement: S4 Fig — (TIF) [file pone.0308111.s005.tif]

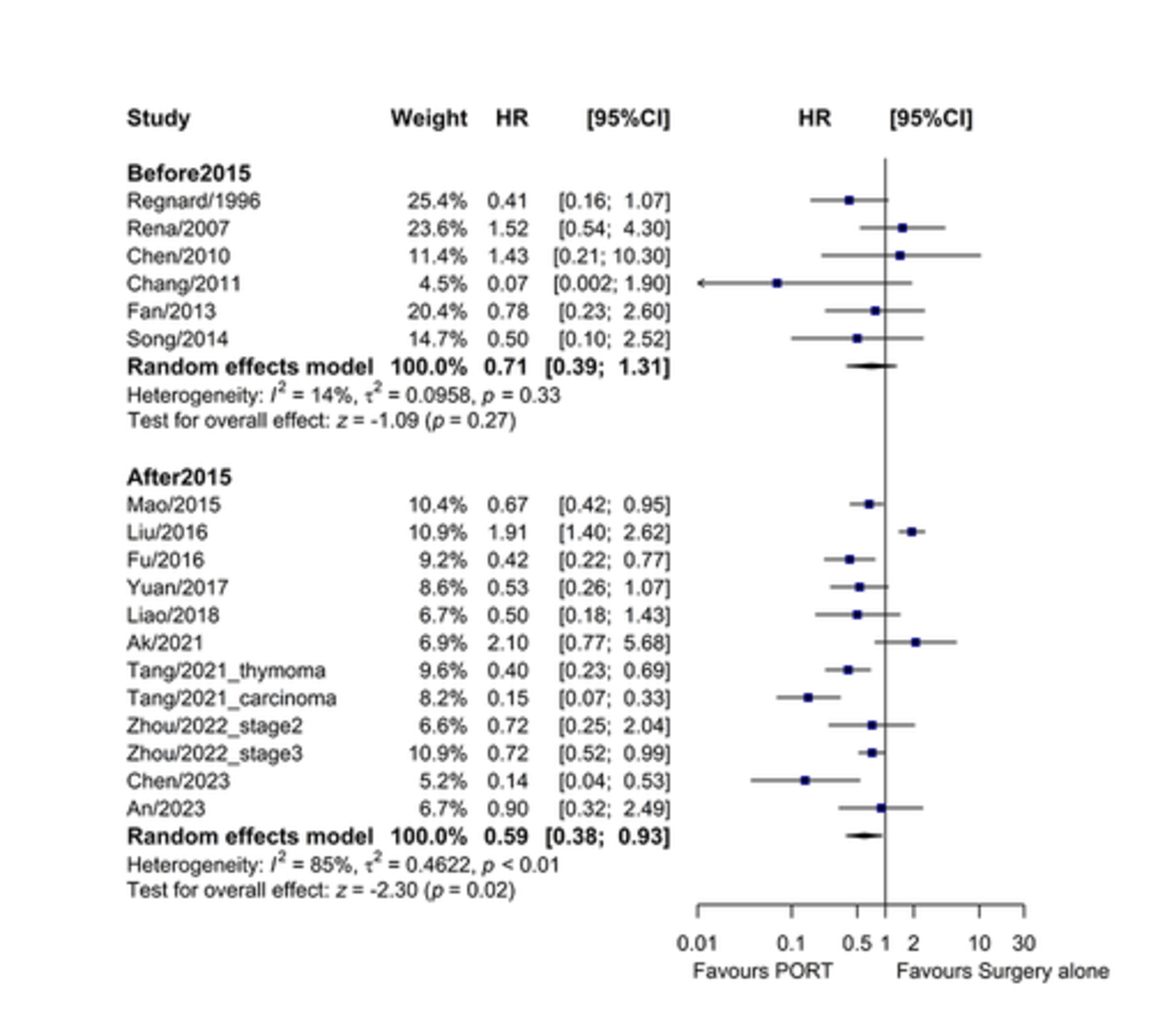

Supplement: S5 Fig — (TIF) [file pone.0308111.s006.tif]

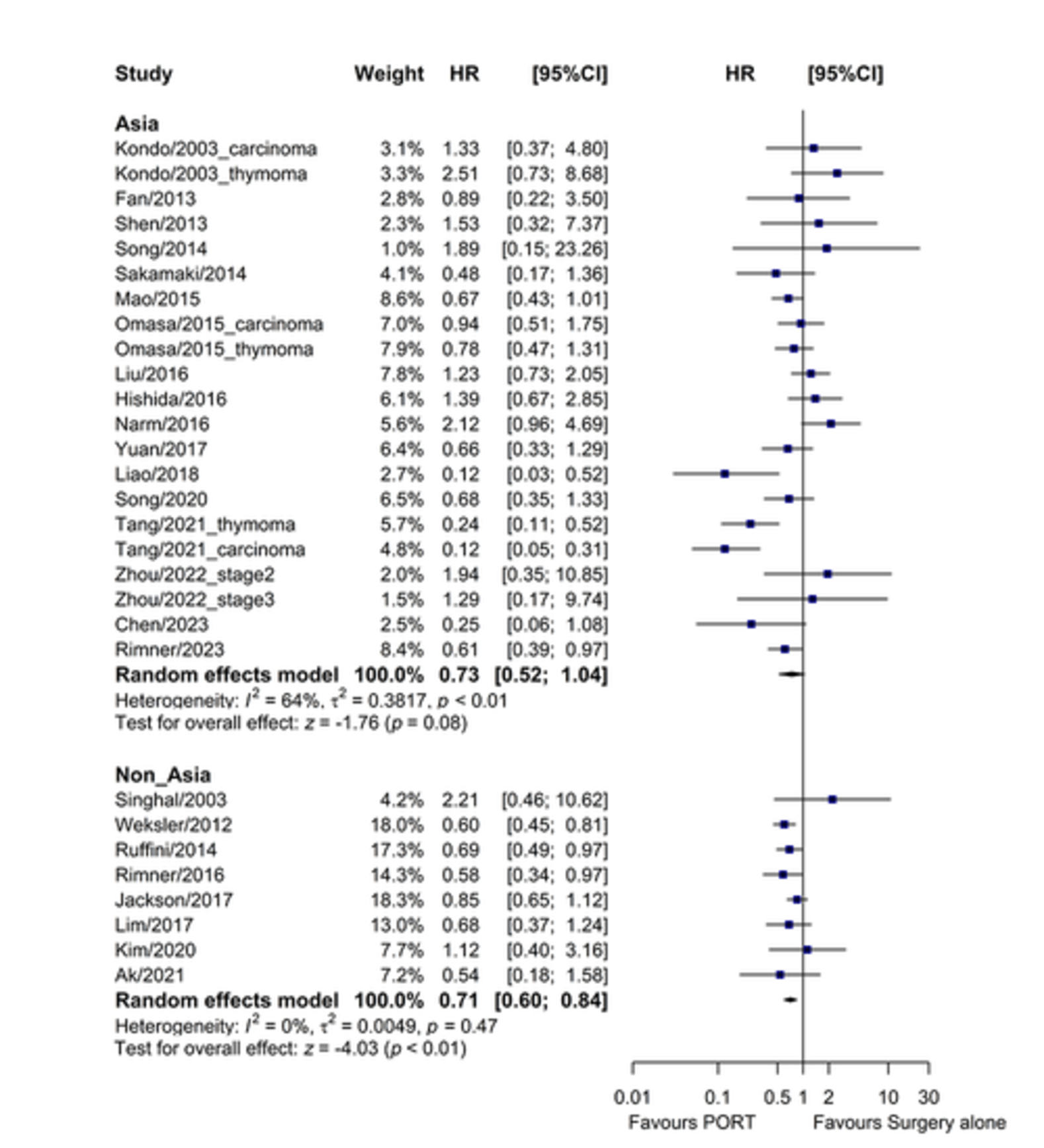

Supplement: S6 Fig — (TIF) [file pone.0308111.s007.tif]

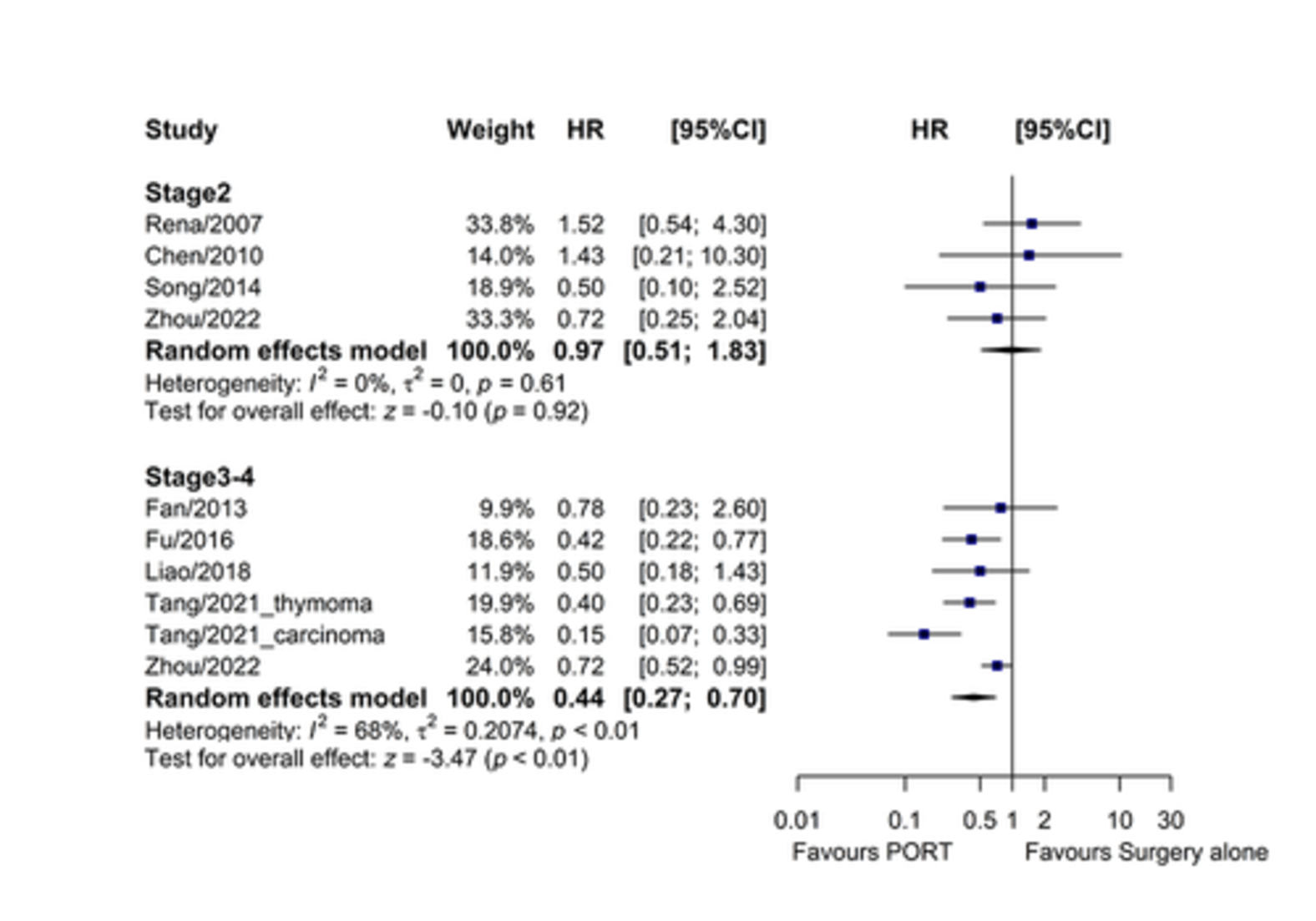

Supplement: S7 Fig — (TIF) [file pone.0308111.s008.tif]

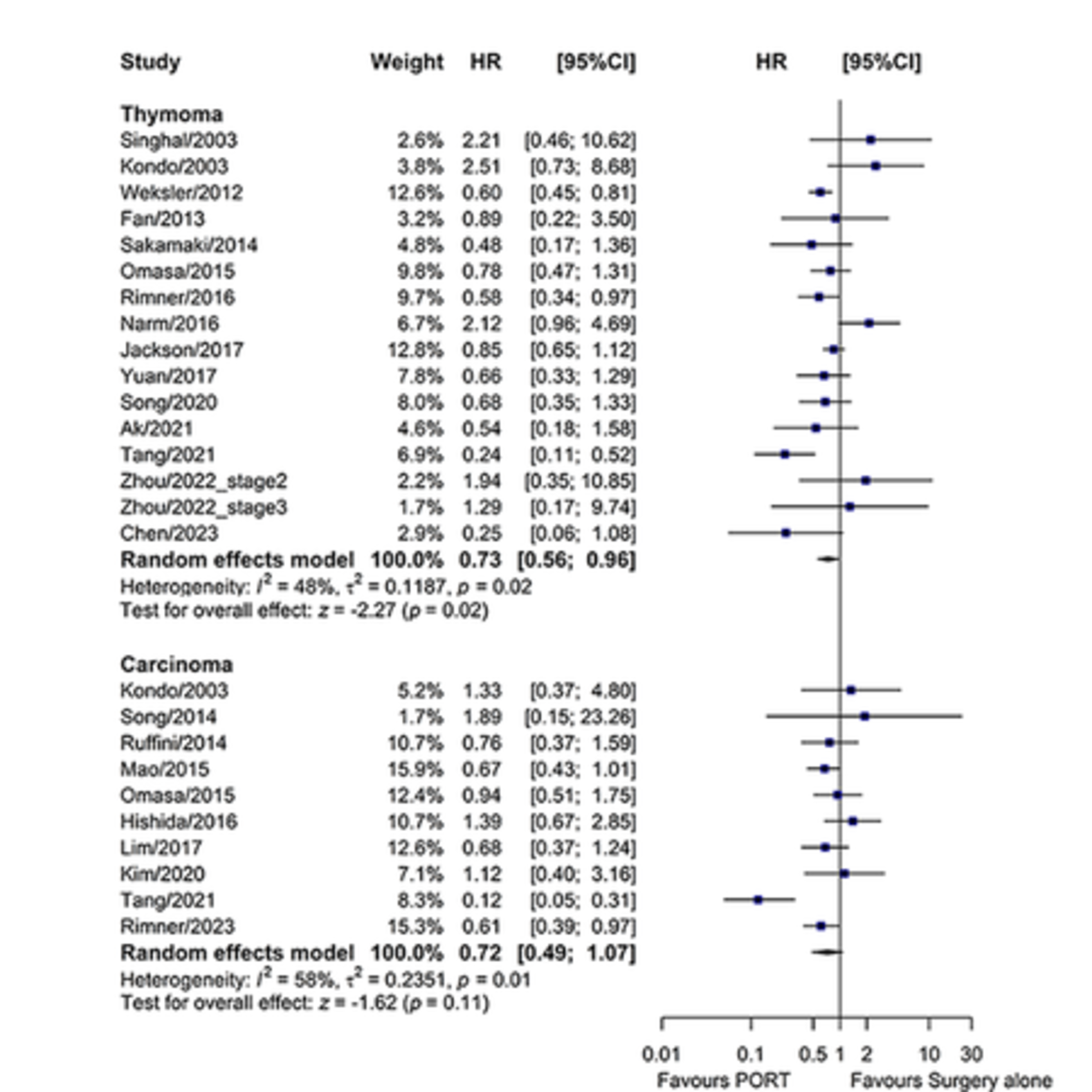

Supplement: S8 Fig — (TIF) [file pone.0308111.s009.tif]

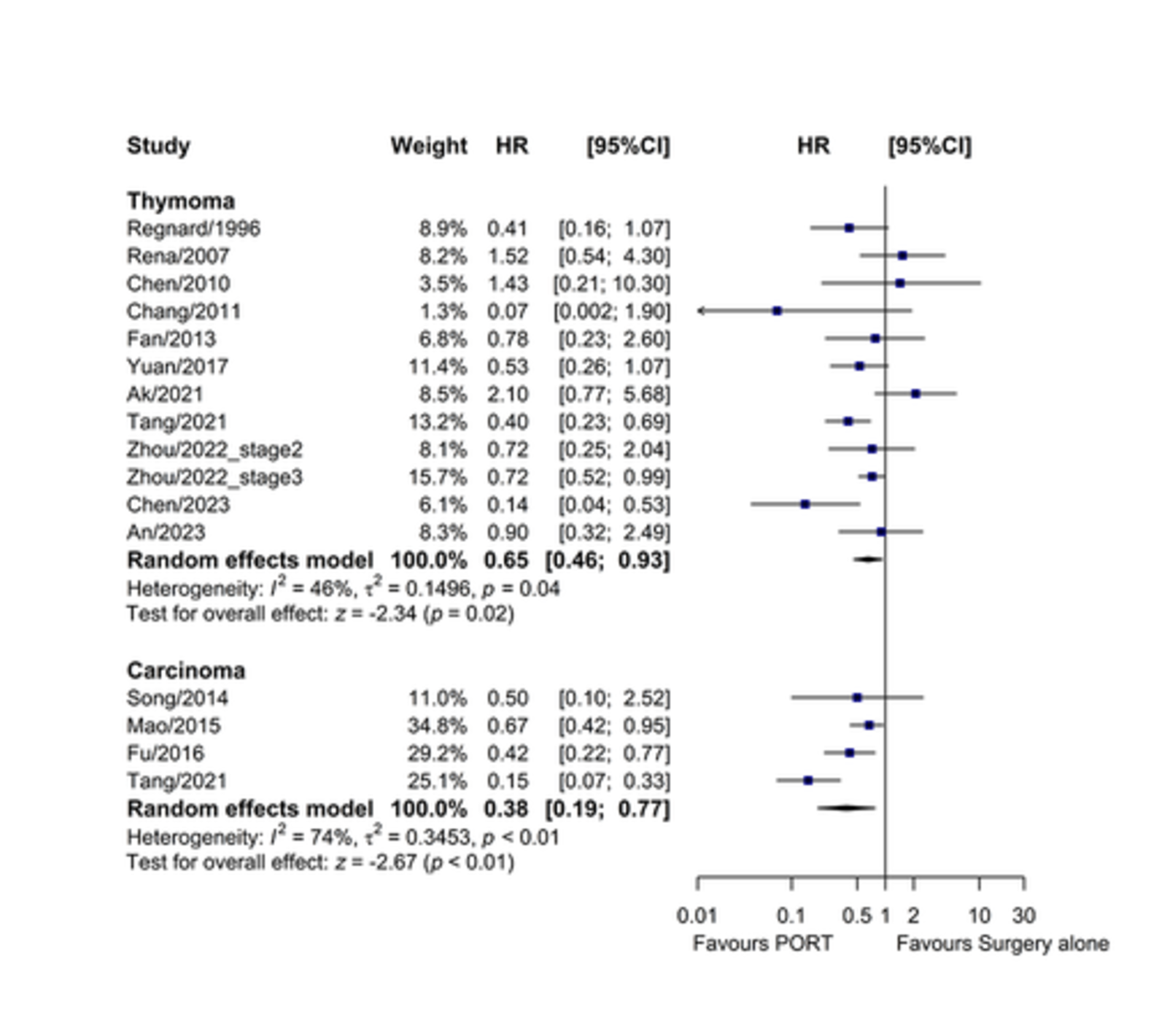

Supplement: S9 Fig — (TIF) [file pone.0308111.s010.tif]

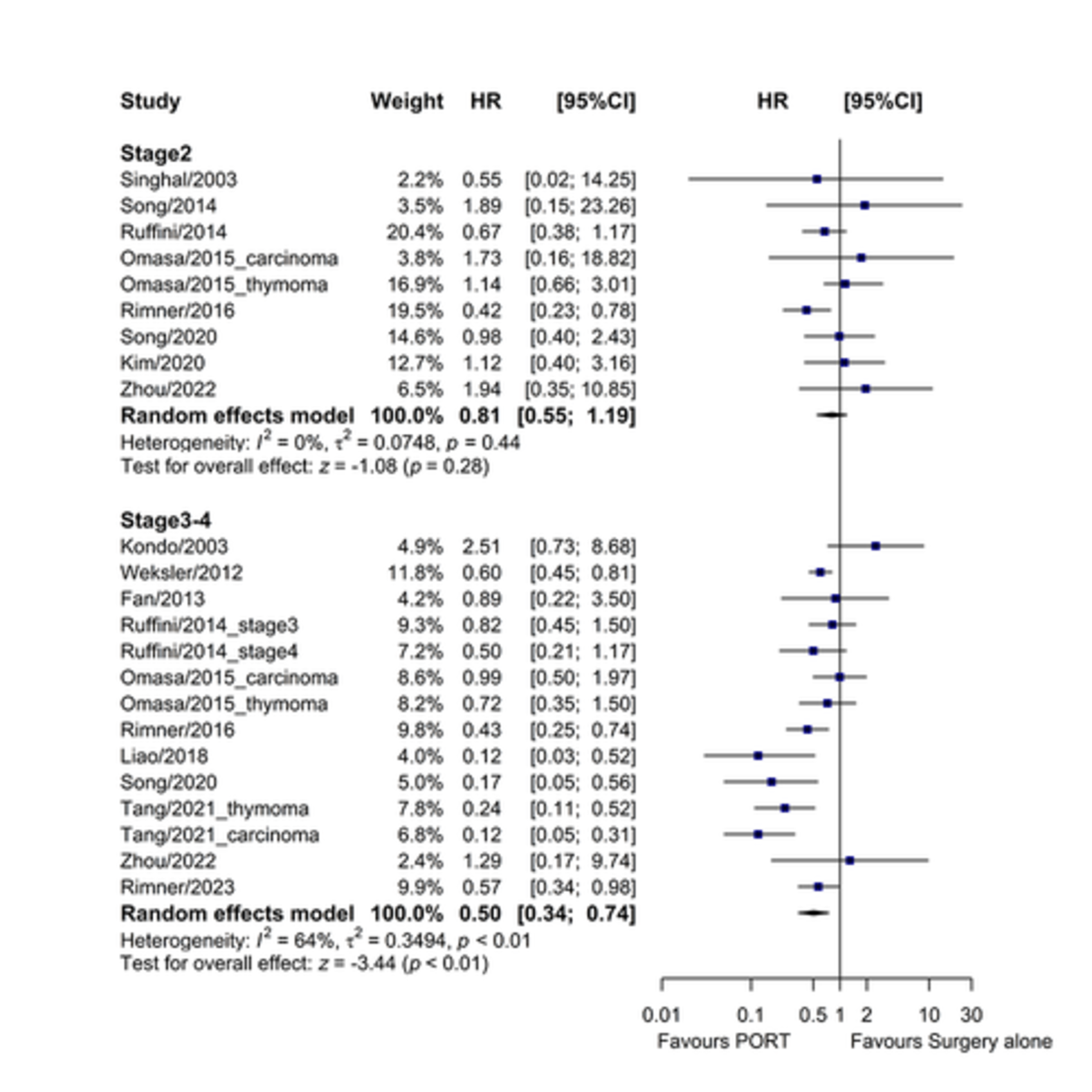

Supplement: S10 Fig — (TIF) [file pone.0308111.s011.tif]

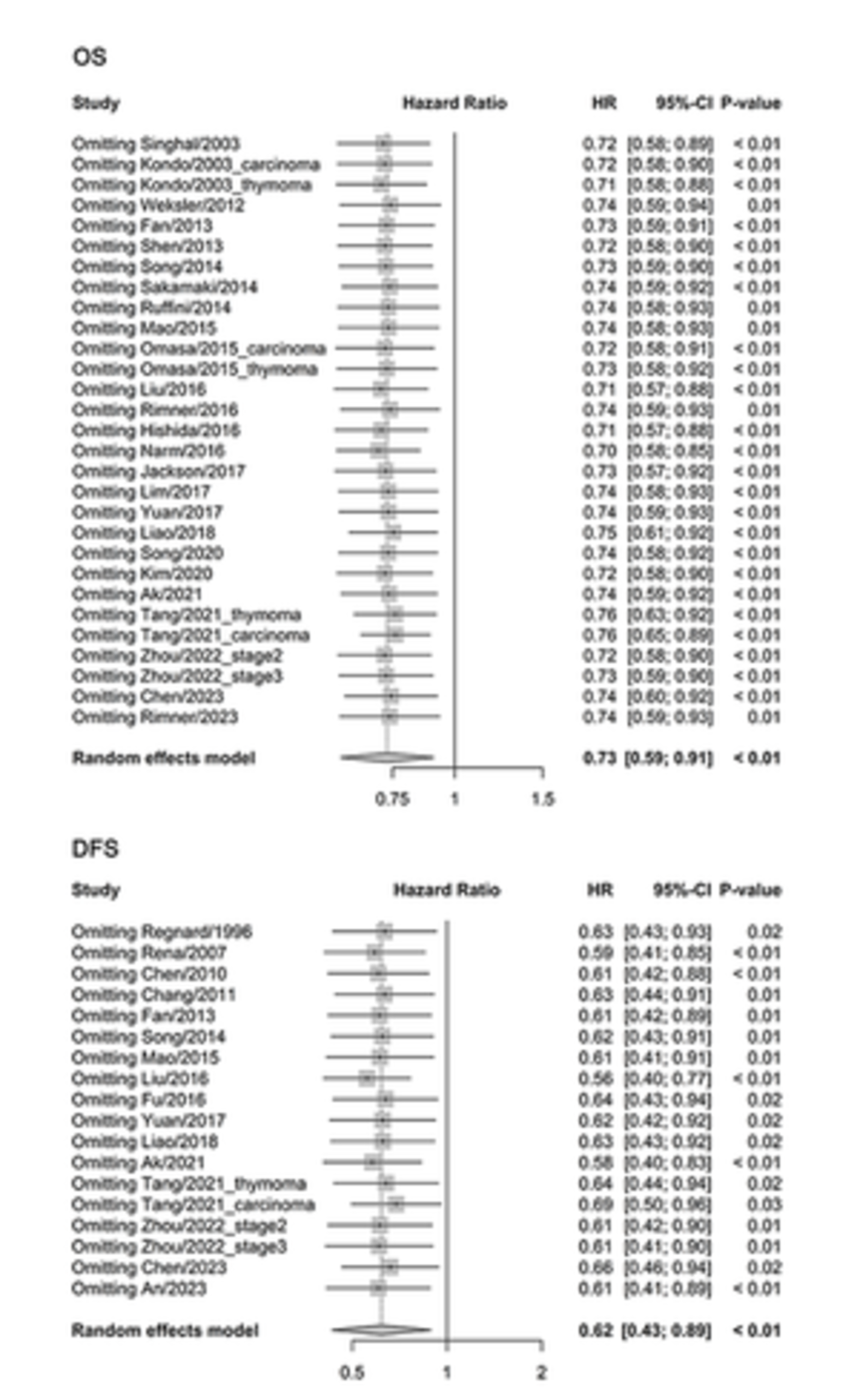

Supplement: S12 Fig — (TIF) [file pone.0308111.s013.tif]
