# Supplementary material for: Postoperative radiotherapy for completely resected thymoma and thymic carcinoma: A systematic review and meta-analysis
Source: PLoS One. 2024 Aug 30;19(8):e0308111. doi: 10.1371/journal.pone.0308111 (PMC11364254; doi:10.1371/journal.pone.0308111)
Supplement: S1 Table — (DOC) [file pone.0308111.s014.doc]

**S1 Table** Search strategy

**a:** Search strategy in PubMed

| # | Query |
| --- | --- |
| #1 | "Thymus Neoplasms"[mh] |
| #2 | Thymic Epithelial Tumors[tiab] OR Thymic Tumor[tiab] OR Thymoma[tiab] OR Thymic Carcinoma[tiab] OR Thymic Cancer[tiab] |
| #3 | #1 OR #2 |
| #4 | Radiotherapy[tiab] OR Radiation[tiab] |
| #5 | Postoperative[tiab] OR Postoperation[tiab] OR Adjuvant[tiab] |
| #6 | Surgery[tiab] OR Surgical[tiab] OR Resected[tiab] OR Resection[tiab] |
| #7 | #4 AND #5 |
| #8 | #6 AND #7 |
| #9 | #3 AND #8 |

**b:** Search strategy in Embase

| # | Query |
| --- | --- |
| #1 | ‘thymic neoplasm’/exp |
| #2 | 'thymic epithelial tumors':ab,ti OR 'thymic tumor':ab,ti OR ‘thymoma’:ab,ti OR ‘thymic carcinoma’:ab,ti OR ‘thymic cancer’:ab,ti |
| #3 | #1 OR #2 |
| #4 | 'radiotherapy':ab,ti OR 'radiation':ab,ti |
| #5 | 'postoperative':ab,ti OR 'postoperation':ab,ti OR 'adjuvant':ab,ti |
| #6 | 'surgery':ab,ti OR ‘surgical':ab,ti OR ‘resected':ab,ti OR ’resection':ab,ti |
| #7 | #4 AND #5 |
| #8 | #6 AND #7 |
| #9 | #3 AND #8 |

**c:** Search strategy in Cochrane Library

| # | Query |
| --- | --- |
| #1 | MeSH descriptor: [thymus neoplasms] explode all trees |
| #2 | (thymic epithelial tumors OR thymic tumor OR thymoma OR thymic carcinoma OR thymic cancer):ti,ab |
| #3 | #1 OR #2 |
| #4 | (radiotherapy OR radiation):ti,ab |
| #5 | (postoperative OR postoperation OR adjuvant):ti,ab |
| #6 | (surgery OR surgical OR resected OR resection):ti,ab |
| #7 | #4 AND #5 |
| #8 | #6 AND #7 |
| #9 | #3 AND #8 |

**d:** Search strategy in Web of Science

|  | # | Query |
| --- | --- | --- |
|  | #1 | TS=("thymus neoplasms" OR "thymic epithelial tumors" OR “thymic tumor” OR "thymoma" OR “thymic carcinoma” OR "thymic cancer") |
|  | #2 | TS=("radiotherapy” OR “radiation”) |
|  | #3 | TS=("postoperative" OR "postoperation" OR "adjuvant") |
|  | #4 | TS=("surgery" OR "surgical" OR “resected” OR "resection") |
| #5 | | #2 AND #3 |
|  | #6 | #4 AND #5 |
|  | #7 | #1 AND #6 |
