# Supplementary material for: Postoperative radiotherapy for completely resected thymoma and thymic carcinoma: A systematic review and meta-analysis
Source: PLoS One. 2024 Aug 30;19(8):e0308111. doi: 10.1371/journal.pone.0308111 (PMC11364254; doi:10.1371/journal.pone.0308111)
Supplement: S2 Table — (DOC) [file pone.0308111.s015.doc]

**S2 Table** Quality assessment of retrospective studies using the Newcastle-Ottawa scale

| **First author/ year** | **Selection** | | | |  | **Comparability** | |  | **Outcome** | | | **Score** |
| --- | --- | --- | --- | --- | --- | --- | --- | --- | --- | --- | --- | --- |
|  | Item 1 | Item 2 | Item 3 | Item 4 |  | Item 5 | Item 6 |  | Item 7 | Item 8 | Item 9 |  |
| Regnard/1996 | - | * | * | * |  | - | - |  | * | * | * | 6 |
| Singhal/2003 | - | * | * | * |  | - | - |  | * | * | * | 6 |
| Kondo/2003 | - | * | * | * |  | - | - |  | * | * | - | 5 |
| Rena/2007 | - | * | * | * |  | * | * |  | * | * | * | 8 |
| Chen/2010 | - | * | * | * |  | * | * |  | * | * | * | 8 |
| Chang/2011 | * | * | * | * |  | - | - |  | * | * | - | 6 |
| Weksler/2012 | * | * | * | * |  | - | * |  | * | * | - | 7 |
| Fan/2013 | - | * | * | * |  | * | * |  | * | * | - | 7 |
| Shen/2013 | - | * | * | * |  | - | - |  | * | * | * | 6 |
| Song/2014 | * | * | * | * |  | - | - |  | * | * | - | 6 |
| Sakamaki/2014 | - | * | * | * |  | - | - |  | * | * | - | 5 |
| Ruffini/2014 | * | * | * | * |  | - | - |  | * | * | - | 6 |
| Mao/2015 | * | * | * | * |  | - | - |  | * | * | - | 6 |
| Omasa/2015 | * | * | * | * |  | * | - |  | * | * | - | 7 |
| Liu/2016 | - | * | * | * |  | * | - |  | * | * | - | 6 |
| Rimner/2016 | * | * | * | * |  | - | - |  | * | * | - | 6 |
| Fu/2016 | * | * | * | * |  | - | - |  | * | * | - | 6 |
| Hishida/2016 | * | * | * | * |  | - | - |  | * | * | - | 6 |
| Narm/2016 | - | * | * | * |  | - | - |  | * | * | - | 5 |
| Jackson/2017 | * | * | * | * |  | - | - |  | * | * | - | 6 |
| Lim/2017 | - | * | * | * |  | * | * |  | * | * | - | 7 |
| Yuan/2017 | * | * | * | * |  | - | - |  | * | * | - | 6 |
| Liao/2018 | * | * | * | * |  | * | - |  | * | * | - | 7 |
| Song/2020 | - | * | * | * |  | * | * |  | * | * | - | 7 |
| Kim/2020 | - | * | * | * |  | * | * |  | * | * | - | 7 |
| Ak/2021 | - | * | * | * |  | - | - |  | * | * | * | 6 |
| Tang/2021 | * | * | * | * |  | - | - |  | * | * | - | 6 |
| Zhou/2022 | - | * | * | * |  | * | - |  | * | * | * | 7 |
| Chen/2023 | - | * | * | * |  | * | - |  | * | * | - | 6 |
| An/2023 | * | * | * | * |  | - | - |  | * | * | - | 6 |
| Rimner/2023 | * | * | * | * |  | * | - |  | * | * | - | 7 |

–, zero point; *, one point. Item 1, representativeness of the exposed cohort; item 2, selection of the non-exposed cohort; item 3, ascertainment of exposure; item 4, demonstration that outcome of interest was not present at start of study; item 5, comparability of cohorts on the basis of the design (study controls for the most important factor); item 6, comparability of cohorts on the basis of the design (study controls for other additional factor); item 7, assessment of outcome; item 8, follow-up long enough for outcomes to occur; item 9, adequacy of follow-up of cohorts.
