# Supplementary material for: Postoperative radiotherapy for completely resected thymoma and thymic carcinoma: A systematic review and meta-analysis
Source: PLoS One. 2024 Aug 30;19(8):e0308111. doi: 10.1371/journal.pone.0308111 (PMC11364254; doi:10.1371/journal.pone.0308111)
Supplement: S3 Table — (DOC) [file pone.0308111.s016.doc]

**S3 Table** GRADE assessment

| Outcome | Design | No. of studies /participants | Risk of bias | Inconsistency | Indirectness | Imprecision | Publication bias | Large effect size | Dose effect | Other consideration | Grade of evidence |
| --- | --- | --- | --- | --- | --- | --- | --- | --- | --- | --- | --- |
| OS | Observational | 26/7929 | Not serious | Serious (I2=55%) | Not serious | Not serious | Not serious | Not present | Not present | Plausible confounding | Low |
| DFS | Observational | 16/3225 | Not serious | Serious (I2=78%) | Not serious | Not serious | Not serious | Not present | Not present | Plausible confounding | Low |
| RFS | Observational | 6/2682 | Not serious | Not serious | Not serious | Not serious | Not serious | Not present | Not present | Not present | Low |
| **Subgroup analyses for OS** | | | | | | | | | | | |
| Adjusted HRs | Observational | 12/4593 | Not serious | Not serious | Not serious | Not serious | Not tested | Not present | Not present | Not present | Moderate |
| Publications before 2015 | Observational | 9/2658 | Not serious | Not serious | Not serious | Not serious | Not tested | Not present | Not present | Plausible confounding | Moderate |
| Publications after 2015 | Observational | 20/4466 | Not serious | Serious (I2=63%) | Not serious | Not serious | Not tested | Not present | Not present | Plausible confounding | Low |
| Asia | Observational | 21/5549 | Not serious | Serious (I2=64%) | Not serious | Not serious | Not tested | Not present | Not present | Plausible confounding | Low |
| Non-Asia | Observational | 8/4042 | Not serious | Not serious | Not serious | Not serious | Not tested | Not present | Not present | Plausible confounding | Moderate |
| Thymoma | Observational | 16/5483 | Not serious | Not serious | Not serious | Not serious | Not tested | Not present | Not present | Plausible confounding | Moderate |
| Thymic carcinoma | Observational | 10/718 | Not serious | Serious (I2=58%) | Not serious | Not serious | Not tested | Not present | Not present | Plausible confounding | Low |
| Stage 2 | Observational | 9/2200 | Not serious | Not serious | Not serious | Not serious | Not tested | Not present | Not present | Not present | Low |
| Stage 3-4 | Observational | 14/1552 | Not serious | Serious (I2=64%) | Not serious | Not serious | Not tested | Yes (HR=0.50) | Not present | Not present | Low |
| **Subgroup analyses for DFS** | | | | | | | | | | | |
| Adjusted HRs | Observational | 8/893 | Not serious | Not serious | Not serious | Not serious | Not tested | Yes (HR=0.48) | Not present | Not present | Moderate |
| Publications before 2015 | Observational | 6/451 | Not serious | Not serious | Not serious | Not serious | Not tested | Not present | Not present | Plausible confounding | Moderate |
| Publications after 2015 | Observational | 12/2774 | Not serious | Serious (I2=85%) | Not serious | Not serious | Not tested | Not present | Not present | Plausible confounding | Low |
| Asia | Observational | 15/2854 | Not serious | Serious (I2=80%) | Not serious | Not serious | Not tested | Not present | Not present | Plausible confounding | Low |
| Non-Asia | Observational | 3/371 | Not serious | Serious (I2=67%) | Not serious | Not serious | Not tested | Not present | Not present | Plausible confounding | Low |
| Thymoma | Observational | 12/1357 | Not serious | Not serious | Not serious | Not serious | Not tested | Not present | Not present | Plausible confounding | Moderate |
| Thymic carcinoma | Observational | 4/353 | Not serious | Serious (I2=74%) | Not serious | Not serious | Not tested | Yes (HR=0.44) | Not present | Plausible confounding | Moderate |
| Stage 2 | Observational | 4/320 | Not serious | Not serious | Not serious | Not serious | Not tested | Not present | Not present | Not present | Low |
| Stage 3-4 | Observational | 6/317 | Not serious | Serious (I2=68%) | Not serious | Not serious | Not tested | Yes (HR=0.44) | Not present | Not present | Low |

Abbreviations: GRADE, Grading of Recommendations, Assessment, Development and Evaluations; OS, overall survival; DFS, disease-free survival; RFS, recurrence-free survival; HRs, hazard ratios.
