# Supplementary material for: Postoperative radiotherapy for completely resected thymoma and thymic carcinoma: A systematic review and meta-analysis
Source: PLoS One. 2024 Aug 30;19(8):e0308111. doi: 10.1371/journal.pone.0308111 (PMC11364254; doi:10.1371/journal.pone.0308111)
Supplement: S4 Table — (DOC) [file pone.0308111.s017.doc]

**S4 Table** Meta-regression analysis

| Group | No. of studies | HR (95%CI) | Meta-regression (Univariate) | | Meta-regression (Multivariable) | |
| --- | --- | --- | --- | --- | --- | --- |
|  |  |  | β (95%CI) | P value | β (95%CI) | P value |
| **OS** | | | | | | |
| **Age (median)** | 14 | - | 0.008(-0.04,0.05) | 0.72 | - | - |
| **Sex (male)** | 18 | - | -0.01(-0.03,0.01) | 0.35 | - | - |
| **Published year** |  |  |  |  |  |  |
| Before 2015 | 9 | 0.81(0.58,1.13) |  |  |  |  |
| After 2015 | 20 | 0.68(0.51,0.90) | -0.29(-0.82,0.24) | 0.29 | -0.59(-1.13,-0.05) | **0.03** |
| **Region** |  |  |  |  |  |  |
| Asia | 21 | 0.73(0.52,1.04) |  |  |  |  |
| Non-Asia | 8 | 0.71(0.60,0.84) | -0.003(-0.48,0.47) | 0.99 | -0.22(-0.66,0.22) | 0.32 |
| **Histological type** |  |  |  |  |  |  |
| Carcinoma | 10 | 0.72(0.45,1.14) |  |  |  |  |
| Thymoma | 16 | 0.73(0.56,0.96) | 0.04(-0.48,0.56) | 0.88 | 0.18(-0.29,0.65) | 0.44 |
| Mixed | 4 | 0.68(0.27,1.70) | 0.02(-0.72,0.77) | 0.95 | -0.09(-0.76,0.59) | 0.80 |
| **Stage** |  |  |  |  |  |  |
| Stage2 | 3 | 1.35(0.59,3.12) |  |  |  |  |
| Stage 3-4 | 7 | 0.45(0.19,1.04) | -1.16(-2.17,-0,15) | **0.03** | -1.55(-2.68,-0.43) | **0.007** |
| Others | 19 | 0.89(0.72,1.10) | -0.54(-1.49,0.41) | 0.26 | -0.65(-1.66,0.36) | 0.21 |
| **DFS** | | | | | | |
| **Age (median)** | 10 | - | 0.05(-0.12,0.22) | 0.58 | - | - |
| **Sex (males)** | 12 | - | -0.01(-0.09,0.07) | 0.78 | - | - |
| **Published year** |  |  |  |  |  |  |
| Before 2015 | 6 | 0.71(0.39,1.31) |  |  |  |  |
| After 2015 | 12 | 0.59(0.38,0.93) | -0.15(-1.04,0.73) | 0.73 | 0.29(-0.76,1.34) | 0.59 |
| **Region** |  |  |  |  |  |  |
| Asia | 15 | 0.56(0.38,0.82) |  |  |  |  |
| Non-Asia | 3 | 1.08(0.40,2.92) | 0.66(-0.32,1.63) | 0.19 | 0.55(-0.58,1.69) | 0.34 |
| **Histological type** |  |  |  |  |  |  |
| Carcinoma | 4 | 0.38(0.19,0.77) |  |  |  |  |
| Thymoma | 12 | 0.65(0.46,0.93) | 0.53(-0.24,1.30) | 0.18 | 0.43(-0.39,1.25) | 0.30 |
| Mixed | 2 | 1.07(0.29,3.94) | 1.13(0.04,2.21) | **0.04** | 1.19(0.11,2.26) | **0.03** |
| **Stage** |  |  |  |  |  |  |
| Stage 2 | 4 | 0.97(0.51,1.83) |  |  |  |  |
| Stage 3-4 | 5 | 0.44(0.24,0.79) | -0.77(-1.87,0.32) | 0.17 | -0.86(-1.99,0.27) | 0.14 |
| Others | 9 | 0.66(0.38,1.15) | -0.34(-1.36,0.68) | 0.51 | -0.49(-1.58,0.60) | 0.38 |

Abbreviations: OS, overall survival; DFS, disease-free survival; HR, hazard ratio.
